# Medical software as a virtual service: A multifaceted approach to telemedicine through software-as-a-service and user-centric features

**DOI:** 10.1177/20552076241295441

**Published:** 2024-12-10

**Authors:** Yanlin Mi, Barry A O’Reilly, Sabin Tabirca

**Affiliations:** 1 School of Computer Science and Information Technology, 8795 University College Cork, Cork, Ireland; 2 SFI Centre for Research Training in Artificial Intelligence, 8795 University College Cork, Cork, Ireland; 3 Department of Obstetrics and Gynecology, 57985 Cork University Maternity Hospital, Cork, Ireland; 4 Faculty of Mathematics and Informatics, 113008 Transylvania University of Brasov, Brasov, Romania

**Keywords:** Telemedicine, software-as-a-service, virtual agents, healthcare accessibility, patient–doctor interaction, medical questionnaires

## Abstract

**Background:**

This article presents medical software-as-a-virtual service platform, a comprehensive telemedicine solution integrating software-as-a-service principles with user-centric features to enhance healthcare service efficiency, accessibility, and patient–provider interaction.

**Methods:**

Medical software-as-a-virtual service employs a multifaceted approach, incorporating a robust questionnaire system for data collection and an empathetic virtual agent module to facilitate nuanced patient–provider interactions. The platform prioritizes privacy and data security through advanced encryption standard encryption and data anonymization, aligning with Health Insurance Portability and Accountability Act standards.

**Results:**

The initial deployment of medical software-as-a-virtual service demonstrates significant improvements in data collection efficiency, patient engagement, and healthcare service quality. The platform’s adaptability is evidenced by its successful application in specialized fields such as radiology and prolapse. User feedback underscores the system’s ease of use and potential for reducing healthcare providers’ workload.

**Discussion:**

Despite its promising outcomes, medical software-as-a-virtual service faces challenges, including the need for in-person treatment and limited virtual agent customization. Short-term improvements aim to enhance appointment scheduling, speech recognition, and agent personalization. Long-term goals include integrating artificial intelligence for diagnostic assistance, Internet of Things for comprehensive remote care, and advanced virtual agents for improved patient interaction.

**Conclusion:**

Medical software-as-a-virtual service emerges as a transformative telemedicine solution, effectively addressing contemporary healthcare challenges. Its continuous evolution, marked by the integration of advanced technologies and user-centered design, holds the potential to reshape the landscape of remote healthcare delivery. Future research will focus on refining platform features and examining the broader impact on healthcare systems and patient outcomes.

## Introduction

In the global healthcare domain, the advent of telemedicine marks a significant shift in the paradigm of medical service delivery. Particularly during the COVID-19 pandemic, telemedicine not only ensured the continuity of medical services but also substantially reduced direct contact between patients and healthcare workers, mitigating the risk of virus transmission.^1,2^ However, while telemedicine has introduced convenience in the provision and reception of medical services, it has also posed a series of challenges, including ensuring data accuracy, protecting patient privacy, optimizing the interaction between patients and healthcare providers, and enhancing the overall quality of medical services.^3^

Data plays a crucial role in modern healthcare, supporting daily medical decisions and forming the basis for personalized treatment and precision medicine. In the telemedicine environment, the effective collection, processing, and analysis of vast amounts of medical data to ensure quality and security become key factors driving the development of remote medical services.^4^ Moreover, as patients’ expectations for the quality of medical services continue to rise, providing an equivalent or superior user experience through telemedicine platforms compared to traditional face-to-face services has become a focal point for healthcare providers.^5,6^

To address these challenges, this study introduces the innovative concept of medical software-as-a-virtual service (MSaaVS). By integrating advanced software-as-a-service (SaaS) models, MSaaVS offers a powerful, flexible, and easy-to-manage platform for healthcare providers and significantly enhances the efficiency and accuracy of data collection while optimizing patient interaction through its carefully designed questionnaire system and virtual agent module. The questionnaire system allows healthcare providers to design and customize questionnaires according to specific needs, and its efficient data processing and analysis capabilities ensure the accuracy and integrity of patient information. Meanwhile, the virtual agent module simulates human interaction, providing a more humanized and empathetic interaction environment that enhances patient engagement and satisfaction. These innovations enable MSaaVS to improve not only the convenience and satisfaction of patients but also the efficiency and quality of the entire healthcare service system.

Although integrating artificial intelligence (AI) technology into MSaaVS is seen as an important pathway to enhance system performance and service quality, the current phase of the study focuses more on enhancing the overall performance of the system through the optimization of core functions such as the questionnaire system and virtual agent module. In the future, with the advancement of technology and the accumulation of practical experience, AI is expected to play a more significant role in MSaaVS, bringing deeper data insights and more intelligent interaction experiences to healthcare providers and patients.^7–9^

**Figure 1.**
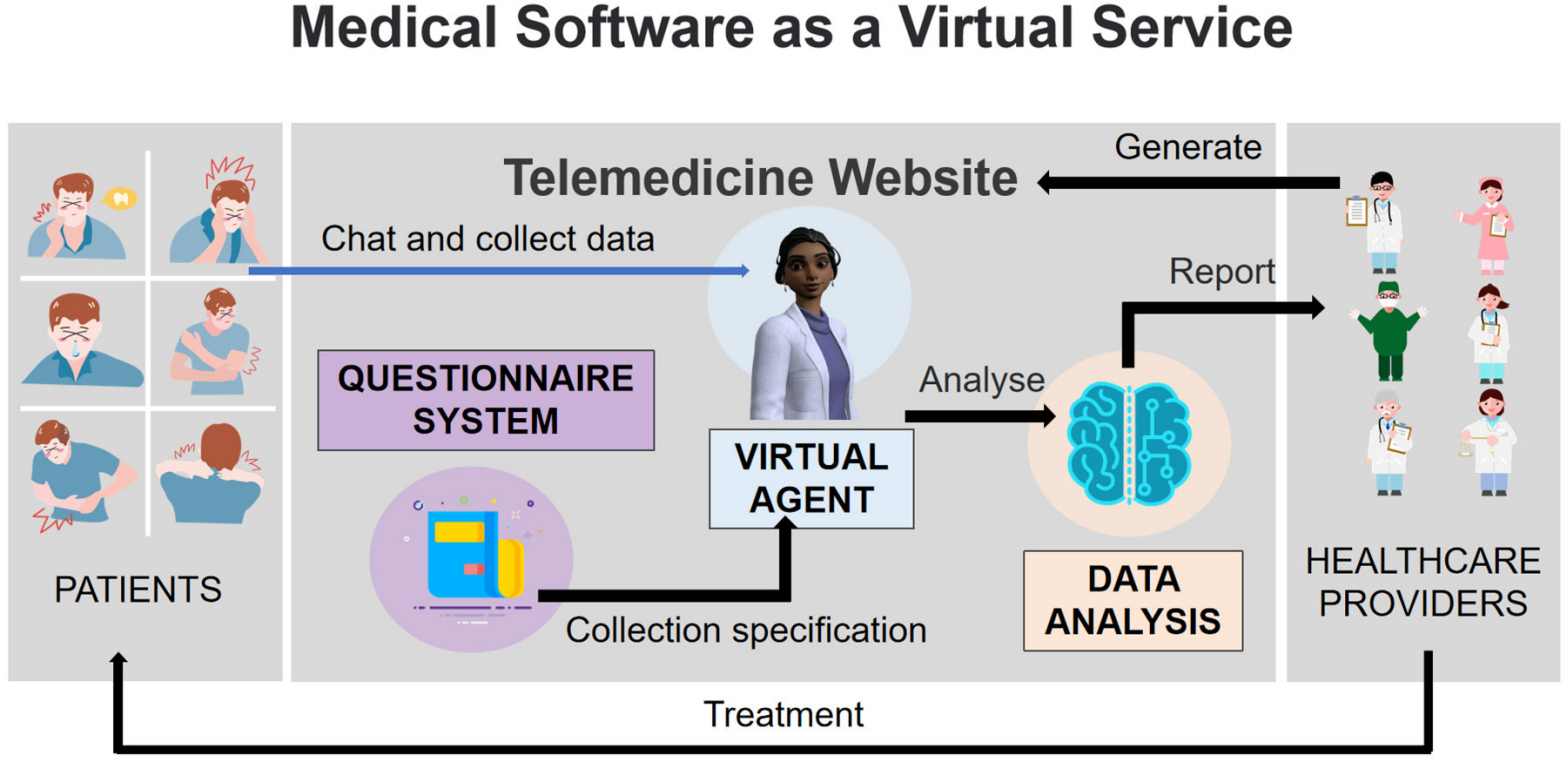
Overview of the medical software-as-a-virtual service (MSaaVS) platform.

As shown in Figure 1, MSaaVS assists healthcare providers in the development of their intelligent telemedicine system. It collects patients’ data and assists in diagnosis. The SaaS platform for healthcare providers can lower development, maintenance, and learning costs, enabling more healthcare providers to deliver services to their patients.^10^ Moreover, MSaaVS does not rely on direct communication between healthcare providers and patients but instead provides a virtual agent that can communicate with patients and collect patient data. The agent allows a healthcare provider to help multiple patients at once and enables patients to request consultations anytime, anywhere. It increases the capacity of healthcare providers to handle a broader range of patients and expands the scope of telemedicine. As a result, healthcare providers from various fields can use MSaaVS, allowing patients to access consultations for most diseases in the future, thus making telemedicine more widespread. MSaaVS is specifically designed to be user-friendly and compatible with web browsers, ensuring that patients and healthcare providers can access the platform and its services regardless of their preferred device.

## Methods

MSaaVS is a SaaS platform designed to offer healthcare providers an innovative telemedicine solution.^11^ Healthcare providers can configure and manage their telemedicine websites through the platform. Compared to traditional healthcare approaches, MSaaVS offers several advantages. Patients can directly access the healthcare provider’s telemedicine website or install the application for essential consultations without waiting to visit a hospital or clinic, resulting in time savings and increased efficiency. Furthermore, healthcare providers can easily create a telemedicine website using MSaaVS without investing significant time and material resources, making it easier for patients to access the right healthcare provider, thus creating a more transparent and fair healthcare market.

The core features of MSaaVS include a questionnaire system and virtual agents, as shown in Figure 1. These features can greatly improve the efficiency and accuracy of telemedicine consultations. The questionnaire system helps healthcare providers create questionnaires, while virtual agents can use pre-created questionnaires to replace healthcare providers for initial consultations and data collection, thus reducing their workload.

### Questionnaire system module

The most effective method for collecting data is through questionnaires, ensuring patients provide consistent data suitable for training machine learning models. The questionnaire system module is one of the essential modules for defining the patient-entered data format.^12^ The module includes the development of question sets and a logic editor, allowing healthcare providers to add and modify the questionnaire’s content using MSaaVS.

Question sets involve adding or removing questions, managing options, and sorting them. Four question types are currently supported: single choice, multiple choice, text input, and color selection. The logic editor provides more flexibility in the questionnaire system, relying on visualization and drag-and-drop operations. Along with typical screen zooming and location dragging, healthcare providers can connect an answer from one question to another to reduce cognitive load. If a specific option is chosen, the patient is directed to the related question, ensuring the questionnaire system’s ease of use and integrity. However, it is essential to consider that patients may find impersonal questionnaires unappealing or unpleasant, particularly if there are many questions. To address this, MSaaVS uses the virtual agent module to handle the questionnaire output and gather responses.

### Virtual agent module

Since the virtual agent always represents the patient’s initial impression of the MSaaVS platform, it can be considered the face of MSaaVS. Healthcare providers can make the most basic customizations to the virtual agent through three configuration items: gender selection, three-dimensional (3D) avatar selection, and voice selection.

**Figure 2.**
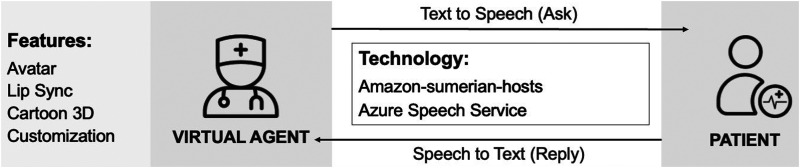
Virtual agent interaction in medical software-as-a-virtual service (MSaaVS).

As a direct contact between the healthcare provider and the patient, the virtual agent collects and communicates information, as shown in Figure 2. The physical characteristics of the virtual agent become the patient’s first impression. The appearance of the virtual agent in MSaaVS is defined by its appearance, speech, facial expressions, and body movements. Due to the performance limitations of browser-based 3D model rendering, the virtual agent’s appearance cannot be entirely realistic like those seen in next-generation games.^13^ However, using a realistic appearance can lead to the “uncanny valley” effect,^14^ which refers to the discomfort and eeriness humans can feel when robots or other human-like objects look almost, but not exactly, like real humans. Therefore, the virtual agent of MSaaVS uses a 3D cartoon model with slightly exaggerated facial features and a head-to-body ratio to reduce this effect while adjusting the patient’s mentality during the consultation through formal dressing and expressions.

Furthermore, the virtual agent must engage in meaningful patient interactions, which require verbal and nonverbal communication. To achieve effective communication, the virtual agent must convert the text from the questionnaire into speech, which current technology can easily accomplish. However, the real challenge lies in ensuring that the virtual agent’s facial expressions and body language are synchronized with the spoken words to create an authentic and distraction-free experience for the patient during the consultation. Moreover, the virtual agent must be able to receive input from the user through various means, such as text, clickable options, or voice commands, to ensure accessibility for all patients. Achieving this requires leveraging advanced natural language processing (NLP) techniques, such as machine learning algorithms and neural networks, which enable the system to comprehend the meaning conveyed by spoken words. For this purpose, MSaaVS relies on Microsoft’s speech-to-text technology, which employs cutting-edge deep learning models to enhance transcription accuracy and ensure optimal performance for the virtual agent.

### Privacy and data security

The implementation of robust security measures and privacy protocols is recognized as a cornerstone of MSaaVS. Due to the sensitive nature of healthcare data, a comprehensive, multi-tiered approach to data security and privacy has been devised, giving priority to both the safeguarding of user information and adherence to regulatory compliance.

One of the critical steps in this procedure is data encryption, both during transit and while stored. The advanced encryption standard (AES) with a key size of 256 bits is employed, which is currently seen as the industry standard and is even used by the US federal government for encrypting classified information.^15^ This powerful encryption method ensures that user data is made unreadable to unauthorized individuals, protecting it from potential breaches.

In addition to encryption, data anonymization techniques are utilized during data processing. Data anonymization is the process of either encrypting or removing personally identifiable information from data sets, so the individuals whom the data describe remain anonymous. This technique not only strengthens privacy but also ensures compliance with the Health Insurance Portability and Accountability Act (HIPAA),^16^ a crucial legal requirement in the healthcare sector.

However, privacy measures don’t stop at data protection. Stringent access controls are implemented to restrict data access to only authorized personnel. This means that even within the system, user data is kept protected and inaccessible to those without the necessary permissions. This layer of security adds another barrier to potential internal breaches.

### Technical implementation

MSaaVS is primarily a software-based solution, leveraging various modern software tools and technologies to deliver its functionality. The core components of the system include:

- Backend services: Utilizing GO and the GIN framework to ensure high performance and scalability.
- Frontend interface: Employing React.js to provide a smooth, responsive user experience.
- Database: Using MySQL and MongoDB to support flexible data models and efficient queries.
- Virtual agent: Integrating TensorFlow.js for NLP, enabling intelligent interactions.
- Security layer: Implementing AES-256 encryption standard and OAuth 2.0 authentication protocol to protect data transmission and storage.
- API integration: Designing RESTful APIs to facilitate integration with other medical systems.
- Data analysis: Incorporating Apache Spark for large-scale data processing and analysis.

While MSaaVS itself does not directly include specific hardware components, it is designed to be compatible with various standard computing devices, including computers, tablets, and smartphones commonly used by healthcare providers and patients. The system’s web-based nature allows for broad accessibility without the need for specialized hardware, aligning with our goal of making telemedicine services widely available and easily adoptable.

### Current development status of MSaaVS

The MSaaVS platform has progressed to an advanced prototype stage, surpassing initial conceptualization and basic functionality. Core functional modules, including the questionnaire system and virtual agent module, have been fully implemented and undergone preliminary deployment and testing in actual healthcare environments. The platform’s development follows an iterative approach, continuously optimizing and refining based on the user feedback and performance metrics.

Currently, MSaaVS enables healthcare providers to create and manage customized telemedicine interfaces, facilitating efficient patient data collection and processing. The system architecture has been designed with future scalability in mind, laying the groundwork for integrating more advanced features such as AI-assisted diagnostics and Internet of Things (IoT) device connectivity.

While MSaaVS has already demonstrated significant practical value, we recognize the ongoing challenges posed by the rapidly evolving field of telemedicine. As such, our development team is actively exploring the application of new technologies, such as the use of augmented reality (AR) in remote diagnostics and the optimization of deep learning-based natural language processing techniques, to further enhance the system’s intelligence and user experience.

## Results

The MSaaVS platform has demonstrated significant potential and tangible benefits in the field of remote healthcare during its preliminary deployment and testing phases. Its core advantage is primarily manifested in the questionnaire system module.

This module has successfully implemented the collection of patient data in a standardized format, which not only lays the foundation for training future machine learning models but also provides reliable decision support for healthcare providers. Currently, the platform has generated 23 distinct questionnaires, each meticulously designed to meet the diagnostic requirements of specific conditions. These questionnaires include, but are not limited to:

- Bladder Function Assessment
- Bowel Function Assessment
- Sexual Function Assessment
- King’s Health Questionnaire (KHQ)
- Pelvic Organ Prolapse/Urinary Incontinence Sexual Function Questionnaire (PISQ-12)
- International Consultation on Incontinence Questionnaire-Urinary Incontinence Short Form (ICIQ-UI)
- Patient Global Impression of Improvement (PGI-I)
- Patient Global Impression of Change (PGI-C)
- Patient Global Impression of Severity (PGI-S)

The level of understanding MSaaVS has for each question depends on the logic and rules input by healthcare providers during questionnaire creation. The system’s intelligent design allows for complex logical branching, ensuring that the virtual agent can dynamically adjust the order and content of questions based on the patient responses. This flexibility enables MSaaVS to simulate the understanding level of trained medical professionals, thereby providing a personalized consultation experience.

For instance, when assessing bladder function, the system can automatically adjust subsequent questions based on the frequency and severity of symptoms reported by the patient, delving deeper into possible causes or complications. This intelligent consultation process not only improves the efficiency of data collection but also provides doctors with more comprehensive and targeted patient information to aid clinical decision-making.

The sufficiency and effectiveness of these questionnaires depend on the specific needs of each healthcare provider and the characteristics of their patient population. The flexibility of MSaaVS allows medical professionals to continuously add and optimize questionnaires as needed. For example, some specialists may regularly update their questionnaire content based on the latest clinical guidelines or research findings to ensure the collection of the most relevant and up-to-date patient information.

It is worth noting that MSaaVS itself does not perform diagnoses, so disease prediction accuracy is not directly applicable to platform evaluation. However, our partner healthcare institutions report that by using the systematized patient data collected through MSaaVS, they have been able to conduct preliminary symptom assessments and risk screenings more efficiently.

**Figure 3.**
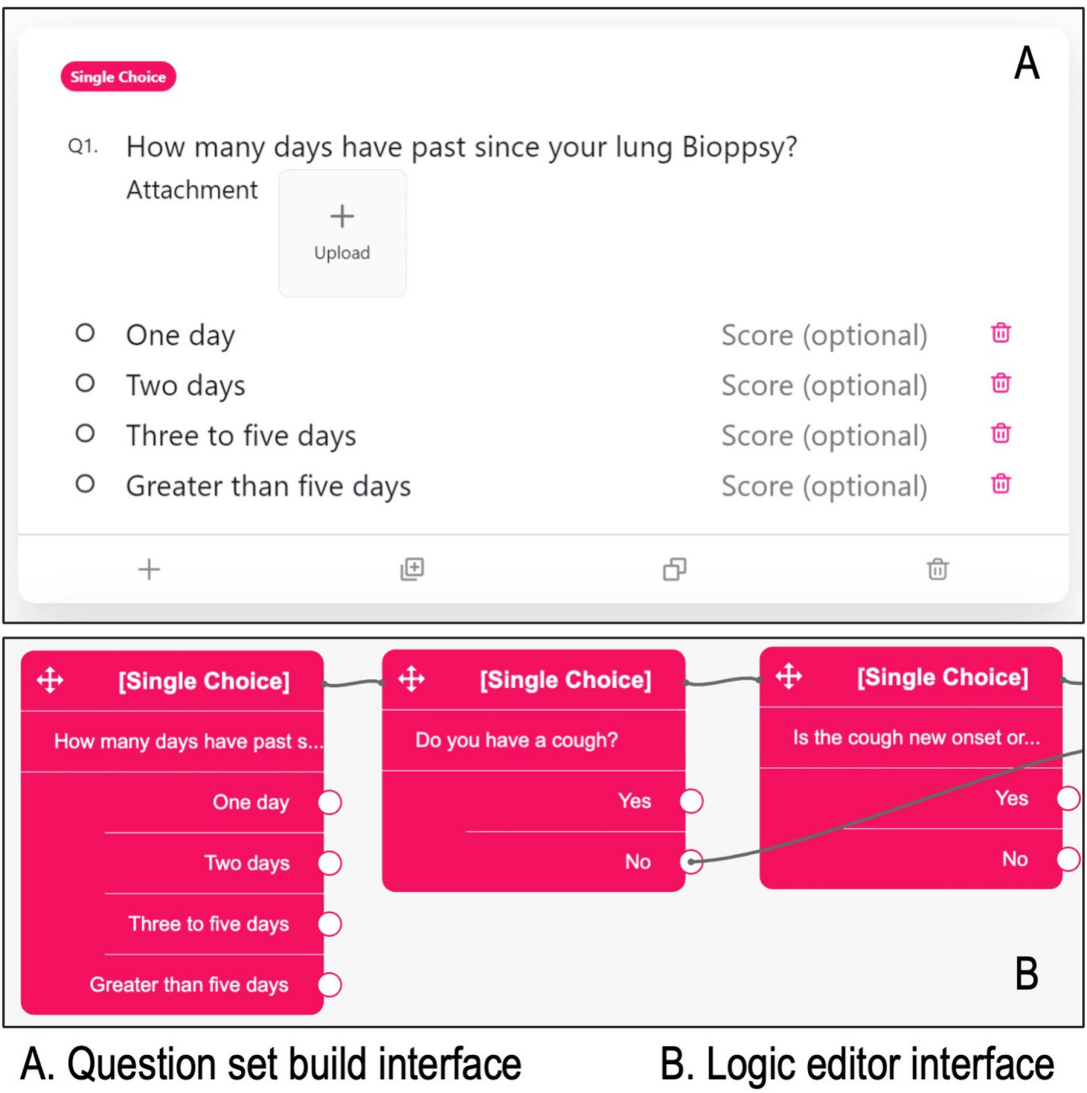
Question set building interface and logic editor interface in medical software-as-a-virtual service (MSaaVS).

As illustrated in Figure 3, the interface of the questionnaire system module prioritizes intuitive user interaction, empowering healthcare providers of varied technical backgrounds to effortlessly create and manage questionnaires. This aspect has been widely acknowledged in user feedback, with numerous medical professionals recognizing MSaaVS for significantly reducing the workload involved in data collection and processing, allowing them to focus more on patient diagnosis and treatment.

**Figure 4.**
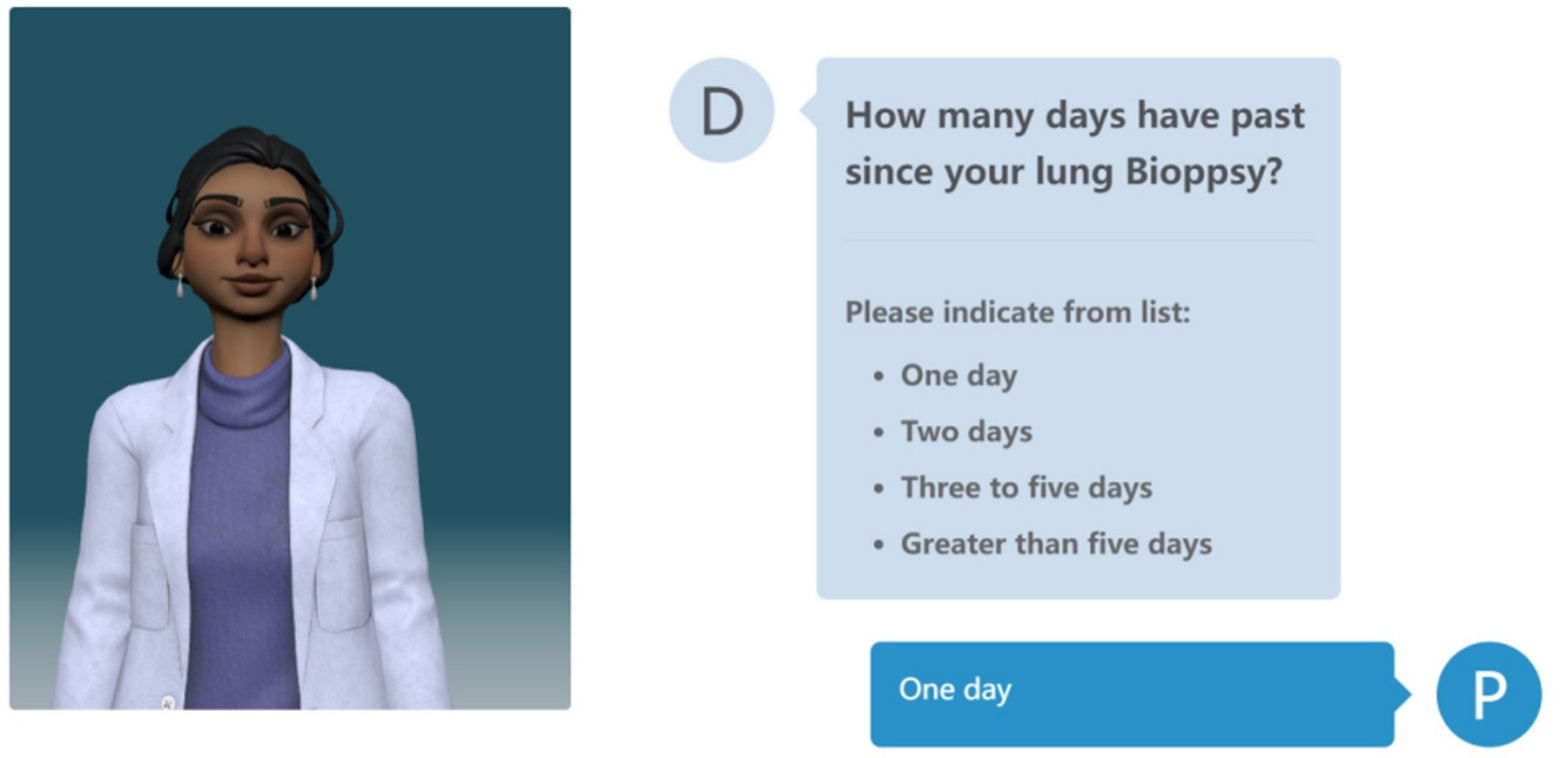
Virtual doctor interface in medical software-as-a-virtual service (MSaaVS).

Furthermore, the virtual agent module, as depicted in Figure 4, has successfully collected responses and offered personalized experiences to patients. The high level of interaction and personalization provided by the virtual agent not only enhances patient satisfaction but also offers healthcare providers a powerful tool to serve patients more efficiently and in a human-centric manner.

During the initial trial phase of MSaaVS, our virtual agent module interacted with a total of 50 patients from various medical specialties. Considering the importance of patient privacy protection, we cannot provide detailed information of individual patients. However, we can present aggregated data on patient interactions and satisfaction rates to demonstrate the effectiveness of the virtual agent.

**Figure 5.**
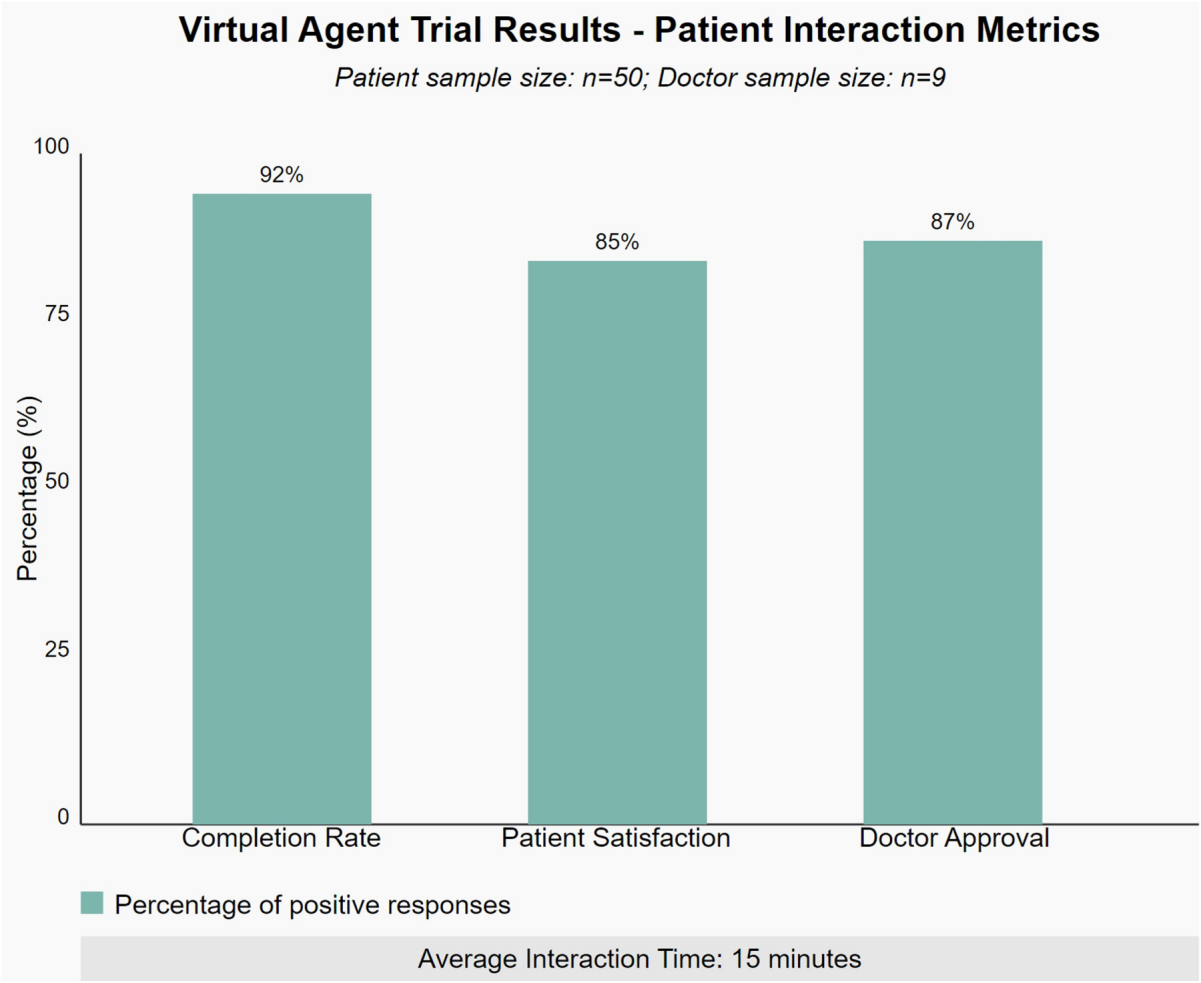
Virtual agent trial results: Patient interaction metrics.

Figure 5 illustrates the key metrics from the virtual agent trials, including average interaction time, patient satisfaction scores, and successful completion rates of questionnaires. The data shows that among the 50 patients who participated in the trial, 92% successfully completed the entire consultation process, with an average interaction time of 15 minutes. Patient satisfaction survey results indicate that 85% of patients were satisfied or very satisfied with the virtual agent’s performance, particularly in terms of accuracy in understanding and responding to questions.

Furthermore, we collected feedback from healthcare providers. Of the nine doctors who participated in the trial, 87% reported that the quality of information collected by the virtual agent was equal to or better than traditional face-to-face initial consultations. This result highlights the potential of MSaaVS in improving healthcare efficiency and quality.

The 85% usability rate of MSaaVS was derived from a comprehensive user satisfaction survey conducted after the trial period. The survey covered multiple aspects of the system, including ease of use, interface intuitiveness, questionnaire clarity, virtual agent interaction quality, and overall user experience.

Table 1 provides a detailed breakdown of the usability metrics that contribute to the overall 85% satisfaction rate. These data are based on survey results from 50 patients.

**Table 1.** Medical software-as-a-virtual service (MSaaVS) usability metrics.

Usability metric	Satisfaction rate (%)
Navigation ease	87
Questionnaire clarity	83
Virtual agent interaction	86
Information input convenience	84
Response speed	88
Overall user experience	85

In specialized medical fields such as radiology and prolapse, MSaaVS has demonstrated its efficacy and applicability. Its flexible design and robust functionality enable it to seamlessly adapt to various medical scenarios and needs, indicating its potential applicability across a broader spectrum of medical fields. It is important to note that the results presented here are based on data collected during the initial testing phase. As the system continues to be optimized and more data is accumulated, the performance and scope of application of MSaaVS are expected to improve further.

Beyond the significant achievements already made, the design of the MSaaVS platform has been envisioned with future development and expansion in mind. With technological advancements and an increase in medical data volume, MSaaVS is expected to integrate more advanced features, such as more intelligent data analytics, more accurate diagnostic support, and more efficient resource management. These potential enhancements and expansions will further solidify the leading position of MSaaVS in the remote healthcare service domain, bringing additional value and convenience to healthcare providers and patients.

## Discussion

### MSaaVS as a telemedicine SaaS platform

**Figure 6.**
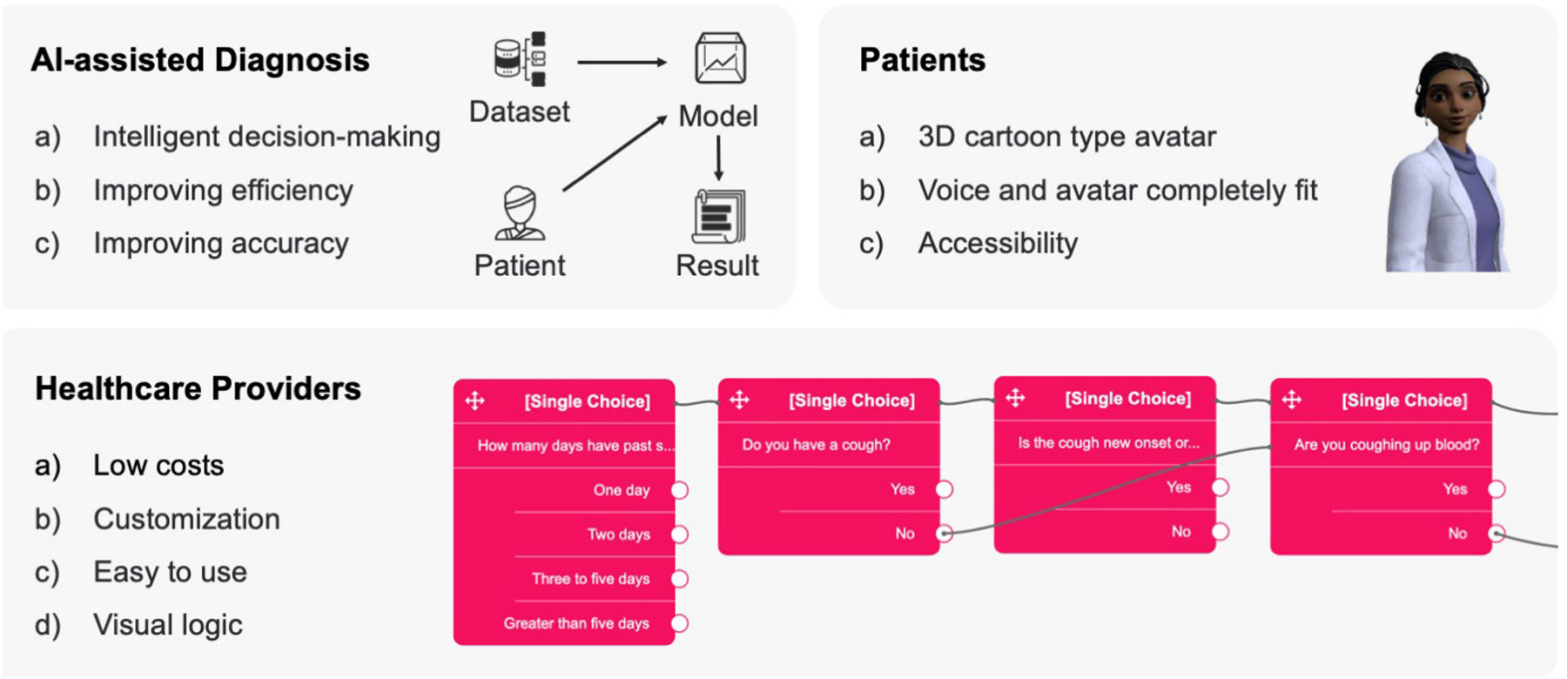
Key features and benefits of the medical software-as-a-virtual service (MSaaVS) platform.

Telemedicine is a rapidly evolving field transforming healthcare by providing patients with increased access to medical care and enabling healthcare providers to deliver care more efficiently and effectively. Despite the increasing interest among healthcare providers to offer remote care services, the cost of developing telemedicine platforms remains a major barrier, limiting the widespread adoption of telemedicine. Additionally, with limited healthcare providers available to serve many patients, long wait times are common and prevent patients from receiving timely medical attention. MSaaVS, a telemedicine SaaS platform, was developed to address these challenges. As shown in Figure 6, MSaaVS can revolutionize healthcare delivery by offering numerous benefits to healthcare providers and patients, enhancing the accessibility and quality of healthcare provision.

For healthcare providers, MSaaVS adheres to the SaaS concept and design principles, helping to lower the costs associated with setting up and operating a telemedicine system. This makes the platform more accessible to healthcare providers, promoting wider adoption of telemedicine. Moreover, the user-friendly interface and intuitive interaction logic of MSaaVS significantly reduce the learning curve for healthcare providers. This level of convenience frees healthcare providers from the burden of technology, allowing them to focus on providing high-quality patient care.

For patients, MSaaVS offers a way to access medical care without the limitations of time and place. Patients can use the platform to consult with healthcare providers from the comfort of their homes without worrying about transportation or wait times. Additionally, the virtual agent can question patients about their symptoms 24 hours a day, making it more convenient for patients to provide information about their health status. With MSaaVS, patients can receive medical care that is both convenient and effective, improving their overall health outcomes.

Overall, MSaaVS provides several advantages to the field of telemedicine, making it an important tool for both healthcare providers and patients. By reducing costs, providing intelligent diagnostic advice, and offering a convenient and user-friendly platform, MSaaVS is helping to revolutionize how medical care is delivered.

### Challenges

Despite the significant contributions of MSaaVS to the healthcare industry, it faces various challenges that require attention. One of the most notable challenges is that consultation is not the only aspect of healthcare. Even if healthcare providers can accurately diagnose patients through teleconsultation, patients may still require in-person treatment at a hospital or medical facility. Additionally, the current approach to remote consultation in MSaaVS is relatively simplistic, limited to surveys that may not meet the needs of patients who prefer personalized and intelligent conversations with their healthcare providers. Another significant challenge faced by MSaaVS is the limited level of customization for its virtual agents. Currently, healthcare providers cannot obtain virtual agents that closely resemble themselves or meet their specific expectations, which can be challenging for those relying heavily on virtual agents to assist with their workload.

### Outlook

Although MSaaVS is already a full-fledged telemedicine platform, our goals go beyond its current state. In the short term, MSaaVS has identified several improvements to enhance its telehealth services. To address the first challenge, MSaaVS plans to add the ability for patients to make appointments online, allowing them to check the business hours of their healthcare provider and schedule an appointment at a specific time that works for them. To tackle the second challenge, MSaaVS intends to enhance its speech-to-text recognition capabilities by incorporating a dictionary of medically relevant terms, helping virtual agents better understand patients’ voices. Finally, to address the third challenge, MSaaVS plans to offer more virtual agent avatars and customizable clothing options to provide a more personalized experience for users.

In the long term, MSaaVS aims to leverage cutting-edge technologies to offer more comprehensive telehealth services. One of the most important goals is to collect enough data from the same questionnaires to train AI models using deep learning techniques, realizing the vision of AI-assisted diagnosis. Additionally, MSaaVS plans to explore the use of IoT, remote care, and remote surgery technologies to eliminate the time and space limitations of visiting a healthcare provider.^17^ These technologies will allow patients to receive full telemedicine services from the comfort of their homes. Furthermore, MSaaVS plans to use NLP techniques and chatbots trained with vast medical datasets to enable virtual agents to gather patient data through more natural communication with patients.^18^ This will provide a more personalized and intuitive experience for patients. Moreover, MSaaVS plans to deploy facial scanning and emotion capture technologies to enable completely customizable anthropomorphic virtual agent avatars, giving healthcare providers more control over their virtual agents’ appearance and improving the overall user experience.

### Limitations and considerations in AI-driven telemedicine

While MSaaVS offers significant advantages in telemedicine, it is crucial to acknowledge certain limitations inherent to AI-driven healthcare systems. One key consideration is the potential for somatization of health problems, where patients may present physical symptoms that are manifestations of psychological distress.

Moreover, certain specific health issues may be overlooked in an AI-driven interaction, particularly those that require subtle interpretation or physical examination. These limitations underscore the importance of positioning MSaaVS as a complementary tool to traditional healthcare rather than a complete replacement for in-person medical evaluation.

To mitigate these risks and enhance the system’s capabilities, we propose the following strategies:

- Regular review and updating of questionnaires to include prompts that may help identify somatization and other complex presentations. This process should involve collaboration with mental health professionals to ensure comprehensive coverage of psychosomatic symptoms.
- Incorporation of open-ended questions that allow patients to express concerns that may not fit into standardized responses. This feature aims to capture nuanced information that might be missed by structured questionnaires.
- Implementation of advanced natural language processing algorithms to analyze free-text responses, potentially identifying patterns indicative of somatization or other complex health issues.
- Clear communication to patients that MSaaVS is a support tool and does not replace comprehensive in-person medical evaluation when necessary. This message should be integrated into the user interface and reiterated at key points during the virtual consultation.
- Ongoing training for healthcare providers on how to effectively use MSaaVS in conjunction with their clinical expertise. This training should emphasize the system’s strengths and limitations, ensuring that providers can critically evaluate the AI-generated insights.
- Development of a hybrid model that combines AI-driven initial assessments with human clinician review, especially for cases flagged as complex or potentially involving somatization.

## Conclusion

In conclusion, this study has demonstrated the potential of the MSaaVS platform as an innovative telemedicine SaaS solution that addresses several critical challenges in the remote healthcare sector. By providing personalized recommendations and a user-friendly interface, MSaaVS aims to enhance accessibility, reduce barriers to entry, and improve overall patient care for healthcare providers and patients alike.

As the demand for remote healthcare services continues to grow, platforms like MSaaVS will play an increasingly important role in ensuring that patients receive timely and accurate diagnoses and treatment. Future research should focus on refining the algorithms and features of the MSaaVS platform, as well as investigating the long-term impacts of such platforms on patient outcomes and healthcare systems. Additionally, exploring the integration of MSaaVS with electronic health records systems and fostering interdisciplinary communication could contribute to better-informed decision-making and improved patient outcomes.
